# Association of obesity with headache among US children and adolescents: Evidence from NHANES 1999-2004

**DOI:** 10.3389/fendo.2022.1072419

**Published:** 2023-01-05

**Authors:** Xin-Xin Bu, Liang-Hua Zhu, Ze-Mu Wang, Chao Lu, Hui Chen, Di Yu

**Affiliations:** ^1^ Department of Paediatric, The First Affiliated Hospital of Nanjing Medical University, Nanjing, China; ^2^ Department of Cardiology, The First Affiliated Hospital of Nanjing Medical University, Nanjing, China; ^3^ Department of Cardiothoracic Surgery, Children’s Hospital of Nanjing Medical University, Nanjing, China

**Keywords:** headache, obesity, cross-sectional study, National Health and Nutrition Examination Survey (NHANES), children and adolescents

## Abstract

**Background:**

Children and adolescents increasingly commonly suffer from obesity and headache. It has been confirmed that there is an association between obesity and headache in adults; however, evidence of such an association in paediatric populations is still controversial. Therefore, this study examined the relationship between obesity and headache among children and adolescents in the US.

**Methods:**

The cross-sectional data of 3948 participants were obtained from the National Health and Nutrition Examination Survey 1999–2004. Weighted logistic regression models were applied to investigate the association between obesity and headache. Subgroup analysis stratified by sex and age was performed to explore the potential difference in the association of paediatric obesity with headache. The performance of paediatric obesity on headache was assessed by receiver operating characteristic (ROC) curve.

**Results:**

The present study involved 3948 participants, of whom 713 (18.1%) had headache. Compared to those without headache, participants with headache tended to be girls and adolescents, have less calcium intake, and have higher levels of body mass index (BMI), C-reactive protein (CRP), serum ferritin and triglycerides (TGs) (all *P* < 0.05). After fully adjusting for potential confounders, the ORs with 95% CIs for headache were 1.03 (0.58–1.54) and 1.25 (0.68–2.30) for overweight and obese participants in comparison with normal-weight controls, respectively, implying no association of paediatric obesity with headache independent of other potential confounding factors. In addition, although higher odds of headache were noted in girls and adolescents (aged 10–17 years), no statistically significant difference was found across any subgroups. The area under the ROC (AUC) of paediatric obesity on headache was 0.634.

**Conclusions:**

In summary, our study indicated that obesity is not associated with headache among US children and adolescents. Further prospective studies with larger sample size are needed to validate our findings.

## Introduction

Paediatric obesity, which has an unfavourable impact on the physical and mental health of children and adolescents worldwide, has emerged as a major health concern over recent decades and is highly related to increased morbidity and mortality in adulthood if timely interventions are not provided (1, 2). Body mass index (BMI) is calculated as weight in kilograms (kg) divided by the square of height in metres (m^2^) and is universally applied in the quantification of the degree of obesity. In adults, BMI is commonly presented as a numerical value to define overweight/obesity (a BMI ≥ 25.0 kg/m^2^ and < 29.9 kg/m^2^ for overweight; a BMI ≥ 30 kg/m^2^ for obesity) (3); however, BMI is not applicable for defining paediatric obesity, as body growth during childhood and adolescence is a dynamic and changeable process and paediatric cut-offs vary depending on age and sex. A paediatric cut-off for overweight and obesity is calculated by multiplying BMI by the 85th percentile and 95th percentile of a patient’s age and sex (4). The prevalence of overweight/obesity in the US paediatric population has been increasing steadily, rising by 2.2% in girls and 4.2% in boys from 1999–2000 to 2003–2004 (5). Globally, the proportion of overweight or obese children and adolescents in developed regions increased to 23.8% of boys and 22.6% of girls in 2013 compared to 16.9% of boys and 16.2% of girls in 1980; similar trends appeared in the prevalence of paediatric obesity in developing regions between 1980 and 2013, with a significant increase from 8.1% to 12.9% in boys and 8.4% to 13.4% in girls (6). Given the rapid expansion of overweight/obese paediatric populations, effective weight management is of utmost importance to ensure the normal growth and physio-psychological fitness of children and adolescents.

Headache, especially migraine-like headache, is a common neurovascular disorder worldwide and is considered the most disabling neurological disease that adversely affects the quality of life of individuals who suffer from it (7). It is currently accepted that the occurrence of headache is intimately related to multiple genetic, environmental, lifestyle, socioeconomic, and nutritional factors (8–13). In addition, several indicators that reflect the health status of the body, such as lipid levels, C-reactive protein (CRP), and blood pressure, have also been demonstrated by previous studies to have an impact on headache (14–16). The primary clinical manifestations of headache can be simply generalized as recurrent attacks of unilaterally or bilaterally located throbbing pain accompanied by a series of concomitant autonomic symptoms, including nausea, vomiting, photophobia, and phonophobia, based on the International Classification of Headache Disorders (ICHD-3), the third edition (17). It has been estimated that 58.4% of children and adolescents under 20 years of age suffer from headache (18). In paediatric populations, the incidence of headache increases with age and peaks in the school-age group, which accounts for approximately 66% of paediatric patients presenting to the emergency department with a chief complaint of headache (19). Once the first attack of headache occurs during childhood and adolescence, headache tends to become a chronic health issue and persist throughout adulthood (20).

Although the link between obesity and headache in adulthood has been robustly acknowledged in the literature, evidence of such an association in paediatric populations remains controversial. A growing body of studies across the world support the hypothesis that a positive correlation between paediatric obesity and headache exists (21–27), while no association between the two disorders has been reported (28, 29). From the perspective of pathophysiology, the underlying mechanisms of the association between obesity and headache may be complex and multidimensional, involving multiple molecular signals at both the central and peripheral levels (30). Since a comprehensive understanding of obesity-associated headache disorder can facilitate the self-management and medical practice of headache among overweight/obese children and adolescents, we examined the relationship between obesity and headache based on data from the National Health and Nutrition Examination Surveys (NHANES).

## Methods

### Study population

**Figure 1:**
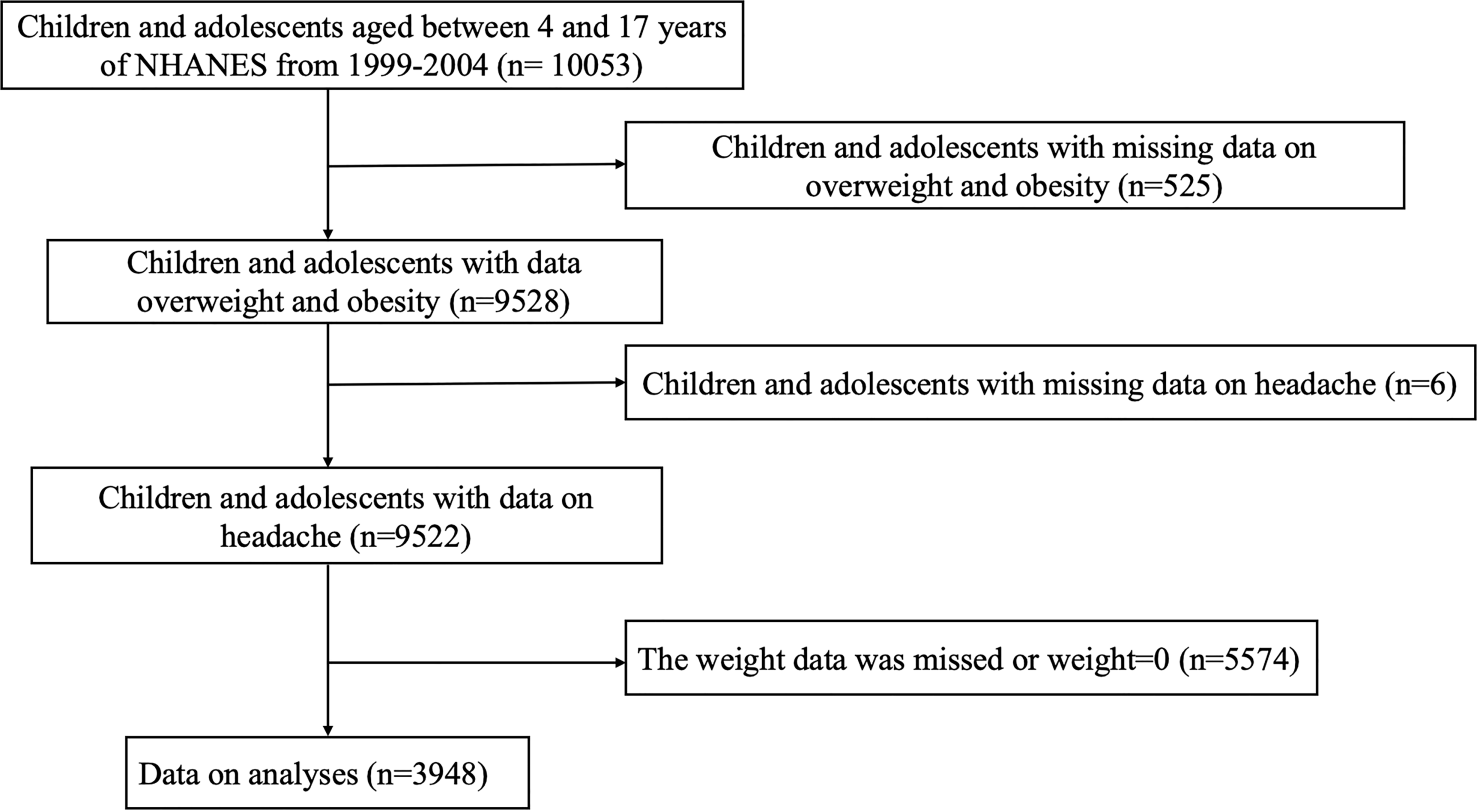
Flow chart of the study population. NHANES: the National Health and Nutrition Examination Survey.

Our study included 10053 children and adolescents (aged 4–17 years) participating in the three consecutive cycles of the NHANES (1999–2004), a large-scale survey that collects cross-sectional data on the health and nutritional status of a nationally representative sample of the US population every two years. The exclusion criteria were as follows: (1) participants without available data on overweight/obesity status (n = 525); (2) participants without available information on headache (n = 6); and (3) participants whose weight data were missing or invalid (n = 5574). Ultimately, a total of 3948 paediatric participants were enrolled for further analyses. Ethical approval to conduct the NHANES 1999-2004 was granted by the NHANES Institutional Review Board. Informed consent was provided by all participants. The study procedures were structured in accordance with the Helsinki Declaration. Detailed information about subject recruitment is illustrated in **Figure 1**.

### Assessment of headache

Diagnoses of headache were made according to the ICHD-3 (17). Participants were considered to have headache if they reported a previous diagnosis of headache by a physician and/or had taken medications for headache and/or answered “yes” to the following question during the interview: “Have you ever experienced headache at least one time in the past three months?”. The classification of headache is composed of four major subtypes: primary headache, secondary headache, neuropathies, and appendix (31). In addition, despite the lack of explicit information regarding the subtypes of headache in the NHANES database, considering the high prevalence of primary headache (including migraine, tension-type headaches (TTHs), trigeminal autonomic cephalalgias (TACs), and other primary headache disorders) in the paediatric population, it is likely that most of the participants included in this study developed primary headache.

### Assessment of overweight/obesity status

Body measurement data, including height and weight, were obtained by trained medical workers during physical examinations following standardized procedures. BMI was calculated by dividing weight (kg) by the square of height (m2) to determine the degree of obesity (3). Child and adolescent obesity was defined based on a previous study (32). In brief, the LMS method was used to construct a centile curve for each dataset based on sex. Then, the centile curves for overweight and obesity by sex passed through the adult cut-off point of a body mass index of 25 kg/m^2^ and 30 kg/m^2^ at 18 years of age, respectively. Based on averaging the centile curves above, we established international cut-off points for overweight and obesity in children and adolescents between the ages of 2 and 18 years.

### Covariates

The covariate selection criteria were based on biological considerations and previously published literature. We designed a standardized questionnaire to collect each participant’s demographic characteristics, including age, sex, race, poverty-income ratio (PIR), and nutritional status. Race was classified as follows: Mexican American, non-Hispanic black, non-Hispanic white, other Hispanic, and other race groups (including multiracial). Economic condition was assessed based on PIR, which was calculated as household income divided by the poverty threshold specific to household size, household age and year (33). Self-reported nutritional status was composed of energy intake, protein intake, carbohydrate intake, iron intake, magnesium intake, and calcium intake.

A mercury sphygmomanometer was used to measure blood pressure in accordance with instructions from the National Center for Health Statistics (August 2000, Available from: http://www.cdc.gov/nchs/data/nhanes/pe.pdf). After 5 min of quiet rest in a chair, three consecutive blood pressure readings were obtained and processed to calculate the average blood pressure. In exceptional situations, the methods for calculating the average blood pressure were adjusted according to the following protocols: (1) The diastolic blood pressure (DBP) reading displaying zero was not incorporated to calculate the average DBP, and the average DBP was recorded as zero only if all DBP readings were displayed as zero; (2) If only one blood pressure measurement was valid, that reading was considered representative of the average blood pressure; and (3) If there is more than one blood pressure reading, the first reading is always excluded from the average. In addition, serum concentrations of ferritin, CRP, total cholesterol (TC), and triglycerides (TGs) were examined by laboratory tests.

### Statistical analysis

Complex sampling designs and sampling weights that are in accordance with the NHANES analytic guidelines were considered in our study (34). All analyses were performed using fasting subsample weights. Based on the formula above, the weights for 1999-2004 are equal to 2/3 the weights for 1999–2002 or 1/3 the weights for 2003-2004. Continuous variables are expressed as weighted means (95% Cis) and weighted medians (IQRs), while categorical variables are expressed as unweighted numbers (weighted percentages, %). When comparing the baseline characteristics between paediatric participants with or without headache, the significant differences were analysed with Student’s t test or a nonparametric test for continuous variables and the χ^2^ test for categorical variables.

An analysis of multivariable logistic regression models was conducted to investigate the relationship between obesity and headache in paediatric populations. A range of regression models (Models 1 to 4) were tested by adjusting potential confounding factors step by step. Model 1 served as the crude model. Model 2 was adjusted for race, PIR, TC, TGs, CRP, and serum ferritin. Model 3 was the main model, which was adjusted for the variables in Model 2 and several additional confounders, including systolic blood pressure (SBP), diastolic blood pressure (DBP) and dietary intake of energy, protein, carbohydrates, iron, magnesium, and calcium. Ultimately, in the fully adjusted model (Model 4), age and sex were further adjusted on the basis of the covariates considered in Model 3. The results are presented as adjusted odds ratios (ORs) and 95% confidence intervals (CIs). The association of paediatric obesity with headache was subsequently assessed through subgroup analyses stratified by age and sex. Receiver operating characteristic (ROC) curve was used to evaluate the performance of paediatric obesity on predicting headache.

The statistical analyses were conducted using R version 4.1.3. Statistical significance was determined when the P value was <0.05.

## Results

### Baseline characteristics

We provide detailed baseline characteristics for all participants in this study (**Table 1**). In the study, no significant differences were found in baseline characteristics between included and excluded participants (**Supplementary Table 1**). We enrolled 3948 participants in the current study, of whom 594 (16.6%) were obese and 2125 (44.5%) were overweight. A total of 18.1% of the sample consisted of participants with headache. Compared to those without headache, participants with headache tended to be girls and adolescents, have less calcium intake, and have higher levels of BMI, CRP, serum ferritin and TGs (all *P* < 0.05).

**Table 1:** Baseline characteristics of the study participants.

	Total	Without headache	With headache	P value
Participants (n)	3948	3235	713	
Age, years [mean(95%CI)]	10.51 (10.31,10.70)	10.14 (9.93,10.34)	12.41 (12.05,12.77)	<0.001
Sex				0.001
Female	1945 (49.46) [44.68,54.24]	1540 (47.66) [45.31,50.00]	405 (58.75) [52.92,64.58]	
Male	2003 (50.54) [45.29,55.80]	1695 (52.34) [50.00,54.69]	308 (41.25) [35.42,47.08]	
BMI, kg/m^2^ [mean (95%CI)]	19.81(19.59,20.04)	19.39(19.17,19.61)	22.00(21.34,22.65)	<0.001
Weight status				<0.001
Normal	1229 (38.91) [34.60,43.22]	1111 (42.57) [40.06,45.08]	118 (20.00) [14.48,25.52]	
Overweight	594 (16.55) [14.43,18.68]	490 (16.66) [15.39,17.93]	104 (15.99) [12.18,19.79]	
Obesity	2125 (44.54) [40.28,48.80]	1634 (40.77) [38.16,43.38]	491 (64.01) [57.37,70.65]	
Race				0.368
Mexican American	1317 (12.31) [9.89,14.72]	1076 (12.37) [9.70,15.04]	241 (11.98) [8.45,15.50]	
Non-Hispanic Black	1276 (14.78) [12.25,17.31]	1033 (14.46) [11.52,17.40]	243 (16.44) [12.12,20.76]	
Non-Hispanic White	1031 (60.77) [52.22,69.33]	867 (61.47) [57.83,65.12]	164 (57.17) [49.33,65.00]	
Other Hispanic	148 (5.44) [3.11,7.77]	113 (5.04) [3.07,7.00]	35 (7.53) [2.29,12.78]	
Other Race	176 (6.70) [4.70,8.70]	146 (6.66) [4.94,8.39]	30 (6.88) [2.49,11.27]	
PIR [median (IQR)]	2.17 (1.13,3.88)	2.22 (1.13,3.93)	1.97 (1.12,3.51)	0.114
CRP (mg/dl) [median (IQR)]	0.03 (0.01,0.11)	0.03 (0.01,0.10)	0.04 (0.01,0.14)	0.024
Serum ferritin (µg/L) [median (IQR)]	29.00 (19.00,43.00)	29.00 (19.00,43.00)	34.00 (20.00,47.00)	0.019
TC (mg/dl) [mean (95%CI)]	162.25 (160.33,164.18)	161.74 (159.58,163.90)	164.68 (161.29,168.08)	0.141
Triglyceride (mg/dl) [median (IQR)]	75.00 (56.00,106.00)	74.00 (55.00,105.00)	79.00 (57.00,116.00)	0.011
Systolic blood pressure (mmHg) [mean (95%CI)]	105.72 (105.06,106.38)	105.53 (104.81,106.25)	106.47 (105.07,107.86)	0.234
Diastolic blood pressure (mmHg) [mean (95%CI)]	60.10 (59.48,60.72)	59.87 (59.20,60.54)	60.98 (59.86,62.10)	0.068
Energy (kcal/day) [median (IQR)]	1943.32 (1512.97,2496.30)	1937.50 (1513.00,2470.47)	1980.48 (1504.68,2619.19)	0.305
Protein intake (g/day) [median (IQR)]	63.69 (47.05,85.87)	63.28 (46.85,85.17)	66.65 (49.21,92.65)	0.106
Carbohydrate intake (g/day) [median (IQR)]	262.08 (204.20,344.88)	262.12 (205.40,343.40)	261.80 (201.31,355.76)	0.862
Iron intake (mg/day) [median (IQR)]	13.16 (9.55,18.31)	13.20 (9.70,18.31)	12.98 (8.88,18.23)	0.331
Magnesium intake (mg/day) [median (IQR)]	207.50 (155.00,278.00)	208.00 (155.50,276.00)	205.50 (150.00,280.50)	0.782
Calcium intake, mg/day [median (IQR)]	864.00 (587.50,1205.00)	871.50 (604.94,1207.00)	785.00 (518.33,1194.00)	0.049

Data are shown as unweighted number (weighted percentage, %) [95% CI], weighted mean (95% CI) and weighted median (IQR). Abbreviations: BMI, body mass index; PIR, a ratio of family income to poverty; TC, total cholesterol; CRP, C reactive protein; CI, conﬁdence intervals; IQR, interquartile range.

### Association of paediatric obesity with headache

**Figure 2:**
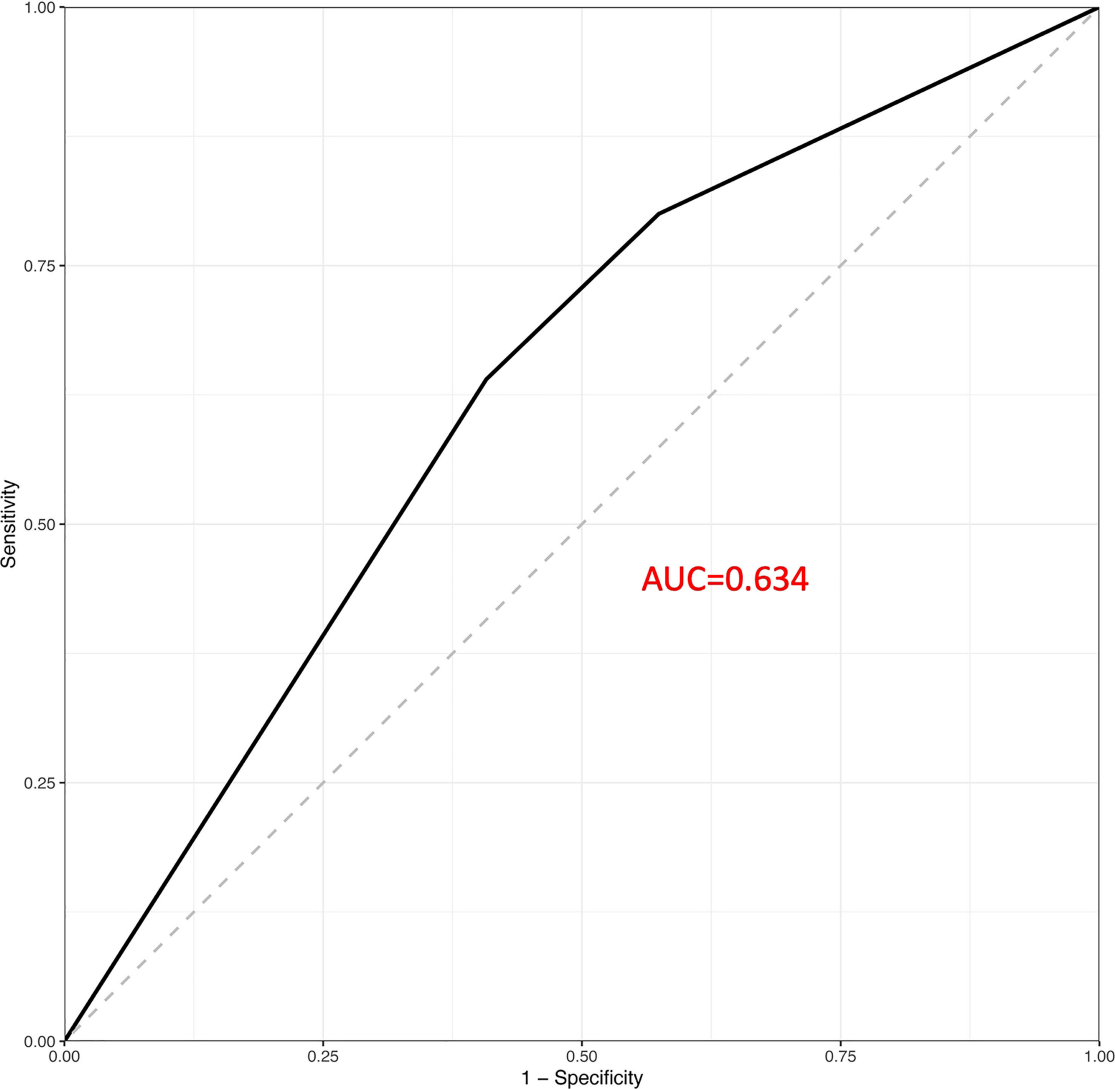
Receiver operating characteristic (ROC) curves.

The results of a sampling-weighted multivariable logistic regression analysis for the association linking obesity with headache among children and adolescents are presented in **Table 2**. Although a positive association was consistently found between paediatric obesity and headache in Models 1–3 (all *P* < 0.05), the ORs with 95% CIs for headache were 1.03 (0.58–1.54) and 1.25 (0.68–2.30) for overweight and obese participants after fully adjusting for potential confounders, including age, sex, race, poverty-income ratio (PIR), TC, TGs, CRP, serum ferritin, SBP, DBP, and dietary intake of energy, protein, carbohydrates, iron, magnesium, and calcium, compared to those of participants with normal weight, respectively (*P* = 0.409), indicating no association between paediatric obesity and headache. In addition, ROC analyses showed that paediatric obesity had a favourable performance to predict headache with an AUC of 0.634 (**Figure 2**).

**Table 2:** Association between obesity and headache in the database from NHANES 1999-2004 (weighted).

Weight status	Unweighted number of total participants	Unweighted number of headache participants	Model 1	Model 2	Model 3	Model 4
			OR (95%CI)	OR (95%CI)	OR (95%CI)	OR (95%CI)
Normal	1229	118	1 (Ref).	1 (Ref).	1 (Ref).	1 (Ref).
Overweight	594	104	2.04 (1.39-3.01) ***	1.98 (1.31-3.01) **	1.31 (0.71-2.44)	1.03 (0.58-1.54)
Obesity	2125	491	3.34 (2.22-5.03) ***	3.38 (2.10-5.43) ***	1.82 (1.01-3.29) *	1.25 (0.68-2.30)
P for trend			<0.001	<0.001	0.038	0.409

Model 1 was crude model.

Model 2 was adjusted for race, PIR, TC, triglyceride, CRP and serum ferritin.

Model 3 was adjusted for race, PIR, TC, triglyceride, CRP, serum ferritin, systolic blood pressure, diastolic blood pressure, dietary intake of energy, dietary intake of protein, dietary intake of carbohydrate, dietary intake of calcium, dietary intake of Iron and dietary intake of magnesium.

Model 4 was adjusted for age, sex, race, PIR, TC, triglyceride, CRP, serum ferritin, systolic blood pressure, diastolic blood pressure, dietary intake of energy, dietary intake of protein, dietary intake of carbohydrate, dietary intake of calcium, dietary intake of Iron and dietary intake of magnesium.

PIR, a ratio of family income to poverty; TC, total cholesterol; CRP, C reactive protein; OR, odds ratio; CI, conﬁdence intervals.

***P < 0.001, **P<0.01, *P < 0.05.

### Subgroup analysis

As shown in **Table 3**, subgroup analysis stratified by age and sex was subsequently performed to detect age and sex differences in the association between paediatric obesity and headache in different subgroups. Numerically, higher odds of headache appeared to be found in overweight or obese girls than in boys (1.23 (0.61–2.49) vs. 0.80 (0.34–1.87) for overweight participants; 1.53 (0.75–3.12) vs. 1.08 (0.48–2.41) for obese participants) and adolescents (aged 10–17 years) than in children (aged 4–9 years) (1.19 (0.68–2.11) vs. 0.22 (0.03–1.65) for overweight participants; 1.50 (0.79–2.83) vs. 0.91 (0.26–3.26) for obese participants); however, there was no statistically significant difference in the association of paediatric obesity with headache across any age and sex subgroups (all P for trend > 0.05).

**Table 3:** Effect of obesity in different sex and age groups (weighted).

Subgroups	Normal (OR)	Overweight [OR (95%CI)]	Obesity [OR (95%CI)]	*P* for trend
Sex				
Female	1(Ref).	1.23 (0.61-2.49)	1.53 (0.75-3.12)	0.240
Male	1(Ref).	0.80 (0.34-1.87)	1.08 (0.48-2.41)	0.717
Age				
4-9 years	1(Ref).	0.22 (0.03-1.65)	0.91 (0.26-3.26)	0.678
10-17 years	1(Ref).	1.19 (0.68-2.11)	1.50 (0.79-2.83)	0.197

Analyses was adjusted for age, sex, race, PIR, TC, triglyceride, CRP, serum ferritin, systolic blood pressure, diastolic blood pressure, dietary intake of energy, dietary intake of protein, dietary intake of carbohydrate, dietary intake of calcium, dietary intake of Iron and dietary intake of magnesium.

PIR, a ratio of family income to poverty; TC, total cholesterol; CRP, C reactive protein; OR, Odds ratio; CI, conﬁdence intervals.

## Discussion

Our cross-sectional study included data from 3948 paediatric participants from a nationally representative US population of the NHANES 1999–2004 for analysis. In the fully adjusted model (Model 4), the ORs with 95% CIs for headache were 1.03 (0.58–1.54) and 1.25 (0.68–2.30) for overweight and obese participants in comparison with normal-weight controls, respectively, implying no association of paediatric obesity with headache independent of other potential confounding factors. In addition, although we failed to detect any statistically significant difference across different age and sex subgroups, more pronounced odds of headache were noted in girls and adolescents, which may suggest that these individuals are more predisposed to headache.

As demonstrated by extensive published literature, obesity during adulthood is strongly correlated with headache, predominantly migraine (35–40). With the high prevalence of obesity and headache during childhood and adolescence, the significance of investigating the association between the two disorders in paediatric populations has been highlighted and has gained more attention than before. Unlike our study, most epidemiological studies support the opinion that paediatric obesity is positively associated with headache (21–26). The impact of paediatric overweight/obesity status on the risk of headache was assessed for the first time in a multi-center, cross-sectional study conducted in Israel, which included 273 children and adolescents (aged 9–17 years) at their first visit to general paediatric and paediatric obesity clinics. Normal-weight girls were 7.7% more likely to develop headache, overweight girls were 14.8% more likely to develop headache, and obese girls were 20.3% more likely to develop headache (*P* = 0.04), whereas this proportion was similar in boys across all three weight groups (13.7% vs. 16.7% vs. 13.2%; *P* = 0.96). A significantly increased risk of hypertension was observed in overweight girls (3.93 (1.28–12.1); *P* = 0.02) and in adolescents 15–18 years of age (2.62 (1.07–6.45); *P* = 0.04) (21). Overall, these results highlight the importance of taking sex and age differences into account in the association between paediatric obesity and headache. In a population-based cross-sectional study performed in Norway, namely, the HUNT study, increased odds of recurrent headache were found to be strongly related to overweight among both girls (1.4 (1.2–1.8); *P* < 0.0001) and boys (1.4 (1.1–1.8); *P* = 0.01) during adolescence (12–19 years of age) (23). Apart from cross-sectional surveys, longitudinal studies may also provide solid evidence underpinning the association between paediatric obesity and headache. In a multicentre retrospective analysis of 913 children and adolescents (aged 3–18 years) presenting to 1 of 7 paediatric headache clinics in the USA, Hershey et al. reported a marked positive correlation between BMI percentiles and headache frequency (*r* = 0.10, *P* = 0.003) as well as between BMI percentiles and disability related to headache (*r* = 0.08, *P* = 0.02) (22). Similar trends exist in two other retrospective studies based on data from paediatric patients who were admitted to either a paediatric neurology clinic or a paediatric outpatient clinic (24, 26). Interestingly, at the 3-month follow-up, a positive association was found between a reduction in BMI percentiles and a reduction in headache frequency in individuals who were overweight at baseline (*r* = 0.32, *P* = 0.01). However, it did not appear to be significant in those with normal weights (r = 0.04, P > 0.05) (*r* = 0.04, *P* > 0.05) (22), which was consistent with the findings from another study that a lower BMI at baseline and an effective reduction in BMI were both predictors of better headache outcomes in overweight/obese adolescents after a 12-month body weight intervention (27). In a prospective cohort study of 3342 Taiwanese school-age adolescents (aged 13–14 years), obesity was found to be merely associated with an elevated risk of chronic migraine (2.43 (1.23–4.80); *P* = 0.011), whereas it was not relevant to chronic tension-type headache (0.51 (0.12–2.16); *P* = 0.358), suggesting that the association of paediatric obesity with headache may partially depend on the subtypes of headache (25). In contrast to those findings mentioned above, some studies together with our study failed to establish an explicit association between the two disorders. Data from 925 children from a retrospective clinic-based study did not support an association between paediatric obesity and headache frequency and showed that the incidence of overweight was not increased in children with chronic migraine or drug overuse (28). Consistently, over 300 headache patients of different ages were analysed by Eidlitz et al., and no association was found between obesity grade and headache attacks in the paediatric age group (29).

Apart from epidemiological studies, the pathophysiological mechanisms underlying the association of paediatric obesity with headache may be more complex and multi-dimensional, involving both central and peripheral pathways that regulate feeding and adipose tissue function and overlapping pathways implicated in the pathogenesis of headache (30). In the central nervous system (CNS), the arcuate nucleus (ARC) of the hypothalamus plays a crucial role in the control of appetite and food intake, predominantly through agouti-related peptide (AgRP)- and proopiomelanocortin (POMC)-expressing neurons that project to the brainstem nuclei, predominantly the nucleus of the solitary tract (NTS) (41). On the other hand, the hypothalamus acts as a key brain region involved in headache pathophysiology, which is widely activated during acute headache attacks (42, 43) and causes patients to present with a series of concomitant signs, including food seeking, sleep disorders and mood fluctuations (44). In addition to the neural circuit basis, various neurotransmitters and neuropeptides derived from the hypothalamus may also be critical molecular effectors linking obesity with headache. Serotonin, primarily through binding to its receptor, the 5-HT_2c_ receptor, has long been implicated in promoting satiety and inhibiting eating behaviours (45). Since chronically low plasma levels of serotonin have been commonly noted in patients with headache (46), serotonin deficiency may be a reasonable explanation for voracious appetite and uncontrollable obesity among these individuals (47). CGRP is the most potent vasodilator in the central nervous system and plays a vital role in the pathogenesis of headache. The mechanisms through which CGRP induces headache may include vasodilation, the activation of meningeal nociceptors, and its action as a central neurotransmitter (48). Patients with headache tend to have higher plasma levels of CGRP (49), and the intravenous infusion of exogenous CGRP can trigger headache attacks (50). Both clinical and experimental evidence has determined the presence of increased plasma levels of CGRP in the context of obesity (51, 52), which may in return be the link between obesity and headache. In addition, whether other hypothalamic neuropeptides with known effects in both feeding control and the modulation of nociceptive processing, such as orexin, oxytocin (OT), neuropeptide Y (NPY), and pituitary adenylate cyclase activating protein (PACAP) (53), are also important players in the association between obesity and headache remains to be elucidated. On the peripheral level, increased adipose tissue, especially visceral adipose tissue, is a hallmark of obesity and acts as a major source of adipokines that are strongly correlated with headache pathogenesis, including leptin, adiponectin, and several pro-inflammatory cytokines. Leptin is a 16 kDa peptide encoded by the obese (ob) gene predominantly regulating appetite and body weight by binding to a specific leptin receptor expressed in the hypothalamus, resulting in reduced food intake and increased energy expenditure (54); the plasma concentrations of leptin are universally upregulated in obese individuals due to leptin resistance (55). According to the latest hypothesis, hyperleptinemia can drive the sensory cortex to generate cortical spreading depression (CSD) as a potential mechanism of headache pathogenesis (56). However, compared to those without headache, in patients with headache, high leptin levels (56, 57) were not consistently noted, and no difference (58) or even low levels (59) were also reported. Adiponectin is another type of adipokine that serves as a critical modulator of energy homeostasis, glucose and lipid metabolism, and the immune response (60). In contrast to leptin as a pro-inflammatory cytokine, adiponectin possesses anti-inflammatory properties, with its levels typically decreasing in the context of obesity (61). Currently, the most widely accepted theory is that obesity, as a chronic low-grade inflammatory state, may reduce the secretion of adiponectin by adipocytes, thereby further perpetuating the inflammation that favours the development of headache. Moreover, pro-inflammatory cytokines, such as interleukin-6 (IL-6) and tumour necrosis factor-α (TNF-α), increase in states of both obesity (62) and acute headache attacks (57), thus contributing to local and systematic inflammation as a prominent feature of headache.

To the best of our knowledge, this is the first study to investigate the association of paediatric obesity with headache in a nationally representative sample of noninstitutionalized US civilians, regardless of the confounding effects of multiple factors. The main strength of our study lies in that the confirmation of such an association is based on the NHANES database, which is a large-scale survey that employs standardized study protocols, strict quality control procedures, and specialized technicians who are well trained to collect and process data, eliminating potential sources of measurement bias to the maximum extent.

However, it should be acknowledged that our study still has some limitations. First, causal relationships are hard to determine owing to the cross-sectional nature of this study. Therefore, robust causal inference should be adopted to verify the current conclusions by longitudinal investigations in the future. Second, although we retrieved and scanned the literature as much as possible for adjustment for confounders, given that headache is a complex and multifaceted disease, there may still exist some factors that play a role in the pathogenesis of headache that remain unidentified. In addition, the NHANES database does not provide explicit information about the subtypes of headache in accordance with the ICHD-3 guidelines, whereas such factors may also have an important impact. In other words, the association of paediatric obesity with headache may be different among patients with different subtypes of headache.

## Conclusion

In summary, our study demonstrated that there was no association between obesity and headache in a nationally representative US paediatric population. Differences in findings between our study and those in the literature highlight several factors that should be addressed, and further prospective studies with larger sample size are warranted to validate our results.

## Data availability statement

The datasets presented in this study can be found in online repositories. The names of the repository/repositories and accession number(s) can be found below: https://www.cdc.gov/nchs/nhanes/index.htm.

## Ethics statement

The studies involving human participants were reviewed and approved by NHANES Institutional Review Board. Written informed consent to participate in this study was provided by the participants’ legal guardian/next of kin.

## Author contributions

DY and X-XB designed the study. X-XB, L-HZ and DY wrote the manuscript. X-XB, L-HZ and Z-MW collected the data, and CL and HC analysed the data. All authors reviewed the manuscript and approved the submitted version.
