# Supplementary material for: Association of obesity with headache among US children and adolescents: Evidence from NHANES 1999-2004
Source: Front Endocrinol (Lausanne). 2023 Jan 5;13:1072419. doi: 10.3389/fendo.2022.1072419 (PMC9849580; doi:10.3389/fendo.2022.1072419)
Supplement: Supplementary file 1 [file Table_1.docx]

**Supplementary table 1 Baseline characteristics of the study participants.**

|  | Total | Include | Exclude | P value |
| --- | --- | --- | --- | --- |
| Participants (n) | 10053 | 3948 | 6105 |  |
| Age, years | 11.28 (4.03) | 11.21 (4.04) | 11.33 (4.03) | 0.154 |
| Sex |  |  |  | 0.144 |
| Female | 5045 (50.2) | 1945 (49.3) | 3100 (50.8) |  |
| Male | 5008 (49.8) | 2003 (50.7) | 3005 (49.2) |  |
| BMI, kg/m^2^ | 21.01 (5.70) | 20.83 (5.57) | 21.13 (5.78) | 0.011 |
| Weight status |  |  |  | <0.037 |
| Normal | 2830 (29.7) | 1229 (31.1) | 1601 (28.7) |  |
| Overweight | 1460 (15.3) | 594 (15.0) | 866 (15.5) |  |
| Obesity | 5238 (55.0) | 2125 (53.8) | 3113 (55.8) |  |
| Race |  |  |  | 0.110 |
| Mexican American | 3338 (33.2) | 1317 (33.4) | 2021 (33.1) |  |
| Non-Hispanic Black | 3197 (31.8) | 1276 (32.3) | 1921 (31.5) |  |
| Non-Hispanic White | 2651 (26.4) | 1031 (26.1) | 1620 (26.5) |  |
| Other Hispanic | 441 (4.4) | 148 (3.7) | 293 (4.8) |  |
| Other Race | 426 (4.2) | 176 (4.5) | 250 (4.1) |  |
| PIR | 1.54 (0.79, 3.01) | 1.53 (0.82, 3.00) | 1.55 (0.77, 3.01) | 0.969 |
| CRP (mg/dl) | 0.04 (0.01, 0.13) | 0.04 (0.01, 0.14) | 0.04 (0.01, 0.12) | 0.254 |
| Serum ferritin (µg/L) | 30.00 (20.00, 46.00) | \| 30.00 (20.00, 45.00) \| \| --- \| | \| 30.00 (20.00, 46.00) \| \| --- \| | 0.808 |
| TC (mg/dl) | 162.86 (30.05) | 162.03 (29.57) | 163.49 (30.40) | 0.029 |
| Triglyceride (mg/dl) | 72.00 (53.00, 100.00) | 72.00 (53.00, 100.00) | 72.00 (54.00, 101.00) | 0.683 |
| Systolic blood pressure (mmHg) | 106.83 (10.40) | 106.81 (10.47) | 106.84 (10.36) | 0.234 |
| Diastolic blood pressure  (mmHg) | 59.62 (11.41) | 60.21 (11.01) | 59.21 (11.66) | <0.001 |
| Energy (kcal/day) | 1950.50 (1497.50, 2505.00) | 1954.13 (1500.00, 2520.27) | 1948.50 (1496.00, 2496.00) | 0.497 |
| Protein intake (g/day) | 65.71 (47.63, 88.25) | 65.22 (47.31, 88.02) | 66.01 (47.95, 88.34) | 0.266 |
| Carbohydrate intake (g/day | \| 261.49 (198.84, 342.06) \| \| --- \| | 261.27 (200.42, 345.38) | 261.62 (197.73, 339.20) | 0.250 |
| Iron intake (mg/day) | 14.79 (10.01, 22.27) | 13.37 (9.51, 18.45) | 16.34 (10.50, 25.59) | <0.001 |
| Magnesium intake (mg/day) | 207.50 (153.00, 278.00) | 204.99 (151.50, 278.11) | 209.00 (154.00, 278.00) | 0.456 |
| Calcium intake, mg/day | 818.97 (543.16, 1179.00) | 809.50 (539.70, 1175.62) | 824.77 (545.03, 1186.30) | 0.320 |

Data are shown as mean (SD), median (IQR), or n (%). Abbreviations: BMI, body mass index; PIR, a ratio of family income to poverty; TC, total cholesterol; CRP, C reactive protein.
